# The impact of Rotavirus mass vaccination on hospitalization rates, nosocomial Rotavirus gastroenteritis and secondary blood stream infections

**DOI:** 10.1186/1471-2334-13-112

**Published:** 2013-03-01

**Authors:** Manuela Zlamy, Sabine Kofler, Dorothea Orth, Reinhard Würzner, Peter Heinz-Erian, Andrea Streng, Martina Prelog

**Affiliations:** 1 Department of Pediatrics, Pediatrics I, Innsbruck Medical University, Anichstr. 35, 6020, Innsbruck, Austria; 2 Department of Pediatrics, Pediatrics II, Innsbruck Medical University, Anichstr. 35, 6020, Innsbruck, Austria; 3 Department of Hygiene, Microbiology and Social Medicine, Division of Hygiene and Medical Microbiology, Innsbruck Medical University, Fritz-Pregl-Str. 3, 6020, Innsbruck, Austria; 4 Department of Pediatrics, University of Würzburg, Josef-Schneider-Str. 2, 97080, Würzburg, Germany

**Keywords:** Rotavirus, Gastroenteritis, Blood stream infection, Children, Universal mass vaccination

## Abstract

**Background:**

The aim of the study was to evaluate the effects of universal mass vaccination (UMV) against rotavirus (RV) on the hospitalization rates, nosocomial RV infections and RV-gastroenteritis (GE)-associated secondary blood stream infections (BSI).

**Methods:**

The retrospective evaluation (2002–2009) by chart analysis included all clinically diagnosed and microbiologically confirmed RV-GE cases in a large tertiary care hospital in Austria. The pre-vaccination period (2002–2005) was compared with the recommended and early funded (2006–2007) and the funded (2008–2009) vaccination periods. Primary outcomes were RV-GE-associated hospitalizations, secondary outcomes nosocomial RV disease, secondary BSI and direct hospitalization costs for children and their accompanying persons.

**Results:**

In 1,532 children with RV-GE, a significant reduction by 73.9% of hospitalized RV-GE cases per year could be observed between the pre-vaccination and the funded vaccination period, which was most pronounced in the age groups 0–11 months (by 87.8%), 6–10 years (by 84.2%) and 11–18 years (88.9%). In the funded vaccination period, a reduction by 71.9% of nosocomial RV-GE cases per year was found compared to the pre-vaccination period. Fatalities due to nosocomial RV-GE were only observed in the pre-vaccination period (3 cases). Direct costs of hospitalized, community-acquired RV-GE cases per year were reduced by 72.7% in the funded vaccination period. The reduction of direct costs for patients (by 86.9%) and accompanying persons (86.2%) was most pronounced in the age group 0–11 months.

**Conclusions:**

UMV may have contributed to the significant decrease of RV-GE-associated hospitalizations, to a reduction in nosocomial RV infections and RV-associated morbidity due to secondary BSI and reduced direct hospitalization costs. The reduction in nosocomial cases is an important aspect considering severe disease courses in hospitalized patients with co-morbidities and death due to nosocomial RV-GE.

## Background

Rotavirus (RV) infections are one of the most frequent causes of gastroenteritis in children worldwide with a major impact on mortality in children <2 years living in developing countries [1-4]. In European countries, mortality attributed to RV infections was estimated to be 1 in 100,000 children <5 years for each year [5-7], with a high burden of nosocomial RV gastroenteritis in the pediatric population [8].

In Austria, about 45,000 episodes of acute RV-associated gastroenteritis (RV-GE) account for approximately 1,400 hospital admissions per 100,000 children [2,5]. Austria, prompted by cost calculations [9], was one of the first European countries to recommend vaccination against RV since 2006 and to subsidize an universal mass vaccination (UMV) program in infants aged between 6 weeks and 6 months with Rotateq (Sanofi Pasteur MSD SNC, Lyon, France; market launch September 2006) between July and December 2007 and with Rotarix (GlaxoSmithKline Biologicals, Rixensart, Belgium; market launch May 2006) between January 2008 and December 2009. Both vaccines are directed against the most important serotypes circulating in Austria, G1P (74.0%), G4P (8.0%) and G3P (1.8%) which were found in samples from hospitalized children due to RV-GE in Innsbruck, Tyrol, and Leoben, Styria [10]. A vaccination coverage of 72 to 87% was documented by surveillance data in 2008 [11]. The hospitalized cases in RV-affected children aged between 3 and 20 months decreased from August 2007 until December 2008 by 74% [11]. A further reduction of RV-GE and some effects of herd protection in older children who were not covered by the UMV because of age limitations were described for 2009 [12]. Both commercially available RV vaccines have a similar efficacy and safety profile [1,13] and have been found to be cost-effective depending on different perspectives and modeling assumptions in some European and developing countries [13-17] with a reduction of all-cause diarrhea-related hospitalizations among children <5 years [18-22].

From the patho-physiological view, RV causes an intestinal epithelium dysfunction in the small intestine. RV-damaged enterocytes are more capable for bacterial invasion causing secondary bacterial infections [23,24]. Only few studies exist thus far which focus on secondary blood stream infections (BSI) as one major and life-threatening complication following RV-GE [24-29].

The retrospective evaluation by chart analysis (2002–2009) focused on all both clinically diagnosed and microbiologically confirmed RV-GE-associated cases in a large tertiary care children’s hospital in Austria. The focus was on RV-GE-associated hospitalizations as the primary outcomes; secondary outcomes were the burdens of nosocomial RV disease and occurrence of secondary BSI as well as direct hospitalization costs for children and their accompanying persons based on the accounts provided by the clearing office of the hospital.

## Methods

### Study design and study population

The retrospective evaluation focused on all cases of RV-GE hospitalized between 1^st^ January 2002 and 31^st^ December 2009 at the Department of Pediatrics, Medical University Innsbruck, a tertiary hospital with additional primary and secondary care functions, covering the area of Tyrol with an average population of 1,277,775 inhabitants between 2002 and 2009. For our analysis, all RV positive stool samples from the Division of Hygiene and Medical Microbiology and the Routine Laboratory of the Department of Pediatrics were matched with the data from the hospital discharge records using the International Classification of Diseases, 10^th^ edition (ICD-10) with search terms “gastroenteritis” (K52.9), “rotavirus” (A08.0), “exsiccosis” (A09) and “blood stream infection” (A41.9) as primary and secondary diagnosis to identify potential RV cases. For inclusion into the study, clinical diagnosis of RV infection had to be confirmed by laboratory results. For included patients a chart review was performed.

### Subgroups of the study population

Groups were separated into a “pre-vaccination period” (January 2002 to December 2005), a “recommended and early funded vaccination period” (intermediate period) (January 2006 to December 2007) and a “funded vaccination period” (January 2008 to December 2009). For determination of the influence of UMV on the age distribution and the hospitalization rates in children with RV infections, children were separated into 5 different age groups according to epidemiological data: infants aged 0–11 months, toddlers aged 12–23 months, children aged 2–5 years, school children aged 6–10 years and children 11–18 years of age. The reasons for age classification are: Most children acquire their first RV-GE before the age of 5 years [1-7]. Severe RV-GE is largely limited to children aged <24 months [1,2]. Vaccinated children aged between 0 and 11 months should have received at least one dose of the vaccine. Toddlers aged between 7 and 24 months are most likely to have been fully vaccinated during UMV [9]. The RV vaccination status could not be collected by chart review. Double-counters and patients admitted more than once due to RV-GE were excluded.

The study was performed according to the principles of the declaration of Helsinki 2008 and the local ethics committee of the Innsbruck Medical University.

### Data collection and definitions

Clinical data from patients included into the study were collected by chart analysis. Cases were defined as nosocomial (hospital-acquired) RV infections if the onset of gastroenteritis-specific symptoms (diarrhea, vomiting) was at least 48 hours after admission to hospital [9] considering an incubation time for RV of 18–36 hours [30] and an admission diagnosis that was not “gastroenteritis”. Gastroenteritis was defined by more than 3 loose stools or watery diarrhea within 24 hours [3] with or without vomiting (particularly in older children), fever and dehydration according to signs given in a scoring system for RV GE [31]. The duration of the hospital stay was defined as the time span between the day of admission and the day of discharge. Nosocomial RV-GE cases were excluded from the analysis of hospital stay durations.

A blood stream infection (BSI) was defined as at least one of the following features: first, a blood culture positive for a pathogen; second, a common pathogen of human skin cultured from two or more blood cultures, both drawn on separate occasions; or third, a common pathogen of the human skin cultured from at least one blood culture in association with signs of a systemic inflammatory response syndrome (SIRS). A SIRS was defined by at least two of the following criteria: elevated body temperature >38°C or hypothermia <36°C, tachycardia, tachypnea, leucocytosis, leukopenia or >10% immature neutrophils according to age ranges. A secondary BSI was defined as a BSI following the clinical symptoms of gastroenteritis associated with laboratory confirmed RV infection more than 48 after onset of RV-associated disease. The present study focused exclusively on secondary BSI.

Hygienic regulations for prevention of nosocomial infections on the ward were not changed during 2002 to 2009. All nurses and doctors with direct contact to the RV-infected patient had to wear over-coats and had to follow a three minutes long hand washing program with Bode Sterillium® Virugard (Paul Hartmann AG, Telgte, Germany) disinfectant solution after patient contact. RV-positive patients and their accompanying persons were cohorted on the ward in separate rooms with own bath rooms and were prohibited to use any facilities on the ward which may have offered the possibility to get in contact with other patients.

### Laboratory confirmation of RV and other pathogens from stool samples

Between 2002 and 2009, all hospitalized patients with gastroenteritis were screened for RV antigen in their stools. For detection of human RV antigen in stool specimen the Pathfinder Direct Antigen Detection System (Kallestad Laboratories, Inc. Austin, Texas) was used till 2005. The sensitivity and specificity of this test system is 84% and 98%, respectively. After 2005, the CerTestRota Card (Biotec, Zaragoza, Spain), an immunochromatographic test for Rotavirus detection in stool specimen was used for routine testing. The sensitivity and specificity of this test system is >99% and 98%, respectively. Routinely, stools were also investigated for additional viral pathogens (Norovirus, Adenovirus) via antigen detection and for bacterial pathogens (*Salmonella*, *Yersinia*, *Shigella*, *Campylobacter spp*. and enterohemorrhagic *Escherichia coli*) via stool cultures according to standard procedures.

### Identification of pathogens in blood culture

From patients showing at least two signs of SIRS, an average of 4 ml blood was drawn for detection of bloodstream pathogens and inoculated into a BacT/Alert PF Pediatric FAN bottle (BioMérieux, Durham, USA). This procedure did not change over the observation period. The bottles were then loaded into a BacT/Alert 3D automated blood culture system (BioMérieux) for a five day protocol with monitoring of carbon dioxide production within each bottle every 10 min. All bottles marked positive were removed from the instrument, and an aliquot was taken for Gram staining and culture on solid media for subsequent analysis. Pathogen identification was performed according to standardized microbial procedures and by VITEK 2 system (BioMérieux).

### Cost calculations

The direct hospitalization costs for children and their accompanying persons are based on the accounts provided by the clearing office of the “Tiroler Landeskrankenanstalten” (TILAK) holding. For the pre-vaccination period the mean of the costs were 870 € per patient per day and for the accompanying person 35 € per day given a proportion of 87.2% accompanying persons in children <6 years (funded vaccination period: 930 €, 38 € and 90.8%, respectively). Children ≥6 years of age are usually not accompanied due to the insurance system which does not refund parents’ costs for the hospital stay together with their child. Nosocomial infections were excluded from cost calculations because of co-morbidity-associated costs which do not allow an approximation of hospitalization costs. Estimated mean of costs per year for community-acquired RV-GE cases were calculated by multiplication of the mean hospital duration (days) (Table 1), mean number of patients per year per age-group (Table 2), mean of direct costs (€) and proportion of accompanying persons in age groups <6 years. Costing procedures have not changed during the study period.

**Table 1:** Clinical and laboratory characteristics of patients with secondary BSI

**Type of RV-GE (number)**	**Mean age (months; range)**	**Sex (female/male)**	**Onset of BSI after RV-GE (hours; range)**	**Underlying disease (number)**	**Pathogens in blood culture (number)**	**Other pathogens in stool samples (number)**	**Mean CRP (mg/dl; range)**
**Community-acquired (6)**	13.9 (11–60)	2/4	149 (48–384)	WPW-syndrome (1)	Staphylococcus aureus (2)	Negative (6)	3.6 (0.2–17.1)
				Bronchitis (1)	Neisseria species (1)		
				None (4)	Enterobacter species (3)		
					Klebsiella species (1)		
					Pseudomonas aeruginosa (1)		
**Nosocomial (14)**	15.6 (0.2–60)	6/8	189 (48–648)	Preterm birth (7)	Staphylococcus aureus (10)	Negative (12)	9.4 (0.3–19.3)
				SOT (2)	Streptococcus pneumoniae (1)	Clostridium difficile (2)	
				ALL (3)	Enterobacter species (2)	Adenovirus (1)	
				Gastroschisis (1)	Enterococcus faecalis (1)		
				Propionacetemia (1)			

Abbreviations: Rotavirus-gastroenteritis (RV-GE); Wolff-Parkinson-White-syndrome (WPW); solid organ transplantation (SOT); acute lymphatic leukemia (ALL); C-reactive protein (CRP).

Preterm birth was defined by gestational age <37 weeks. In some patients more than one pathogen was found. There was no significant difference between the community-acquired and nosocomial RV-GE groups.

**Table 2:** Reduction of RV-GE in age groups from the pre-vaccination period to the funded vaccination period

	**Pre-vaccination period (2002–2005)**		**Intermediate period (2006–2007)**		**Funded vaccination period (2008–2009)**		**Reduction**^**a**^
	**Numbers of patients (%)**	**Mean numbers/year (95% CI)**	**Numbers of patients (%)**	**Mean numbers/year (95% CI)**	**Numbers of patients (%)**	**Mean numbers/year (95% CI)**	**Decreased by (%)**
**0–11 mos**	360 (35.1)	90.0 (80.2–99.8)	113 (30.4)	56.5 (44.7–68.3)	22 (16.4)	11.0 (2.7–19.3)	87.8
**12–23 mos**	300 (29.2)	75.0 (62.8–87.2)	125 (33.6)	62.5 (37.6–87.4)	55 (41.1)	27.5 (7.4–47.6)	63.3
**2–5 yrs**	254 (24.8)	63.5 (50.8–76.2)	110 (29.6)	55.0 (36.5–73.5)	48 (35.8)	24.0 (11.5–36.5)	62.2
**6–10 yrs**	76 (7.4)	19.0 (15.1–22.9)	19 (5.1)	9.5 (8.3–10.7)	6 (4.5)	3.0 (1.6–4.4)	84.2
**11–18 yrs**	36 (3.5)	9.0 (4.1–13.9)	5 (1.3)	2.5 (1.8–3.4)	3 (2.2)	1.0 (0.8–2.2)	88.9
**All**	1026 (100)	256.5 (222.2–290.8)	372 (100)	186.0 (119.5–252.5)	134 (100)	67.0 (22.7–111.3)	73.9

Abbreviations: *mos* months, *yrs* years, *CI* confidence interval.

^a^Reduction is calculated between mean numbers per year of the pre-vaccination period and the funded vaccination period (decrease in percentage).

### Calculation of hospitalization rates

The Innsbruck hospital covers approximately 70% of all pediatric RV-GE hospitalizations in Tyrol. estimated hospitalization rates were calculated for the pre-vaccination, the intermediate and funded vaccination period using the draw area of Tyrol for patients 0–18 years for the different periods (pre-vaccination period: 652,557; intermediate period: 319,825; funded vaccination period: 305,393) [31] as denominator for calculation of incidence rates.

### Statistical analysis

Statistical analysis was performed with SPSS Version 18.0 (Chicago, IL). Non-parametric Mann-Whitney-U test was used to compare mean hospitalizations per year between the pre-vaccination and the vaccination period. Pearson’s Chi-square test was used to analyze difference in dichotome variables. A p < 0.05 was defined statistically significant.

## Results

### Effectiveness of RV vaccination

During the study period, a total number of 3,090 RV-positive stool samples were collected. After excluding double-counters, 2,533 remaining RV-positive cases were matched with the documented ICD-10 discharge codes. A total of 1,001 RV-positive cases were excluded as there was no clinical diagnosis of RV-GE in these patients.

**Figure 1:**
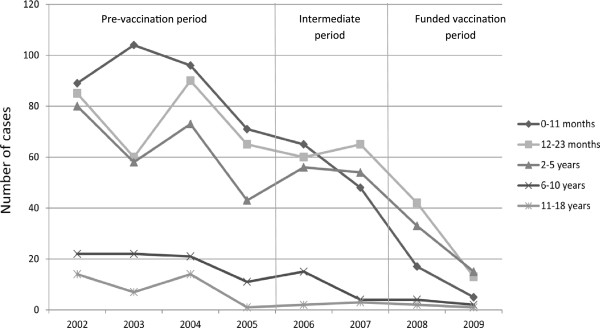
Numbers of hospitalized RV-GE cases.

The mean age of the RV-GE afflicted 1,532 patients (809 male, 723 female) was 2.3 years (median 1.4 years; range 3 days–16.5 years) (Table 2). A significant reduction of hospitalized RV-GE cases per year could be observed between the pre-vaccination and the funded vaccination period (p < 0.001) (Table 2, Figure 1). The absolute number of mean cases per year decreased most strongly in children below 6 years of age (Figure 1). The proportional reduction of hospitalized RV-GE cases per year and age group was most pronounced in the age groups 0–11 months, 6–10 years and 11–18 years (Table 2).

**Figure 2:**
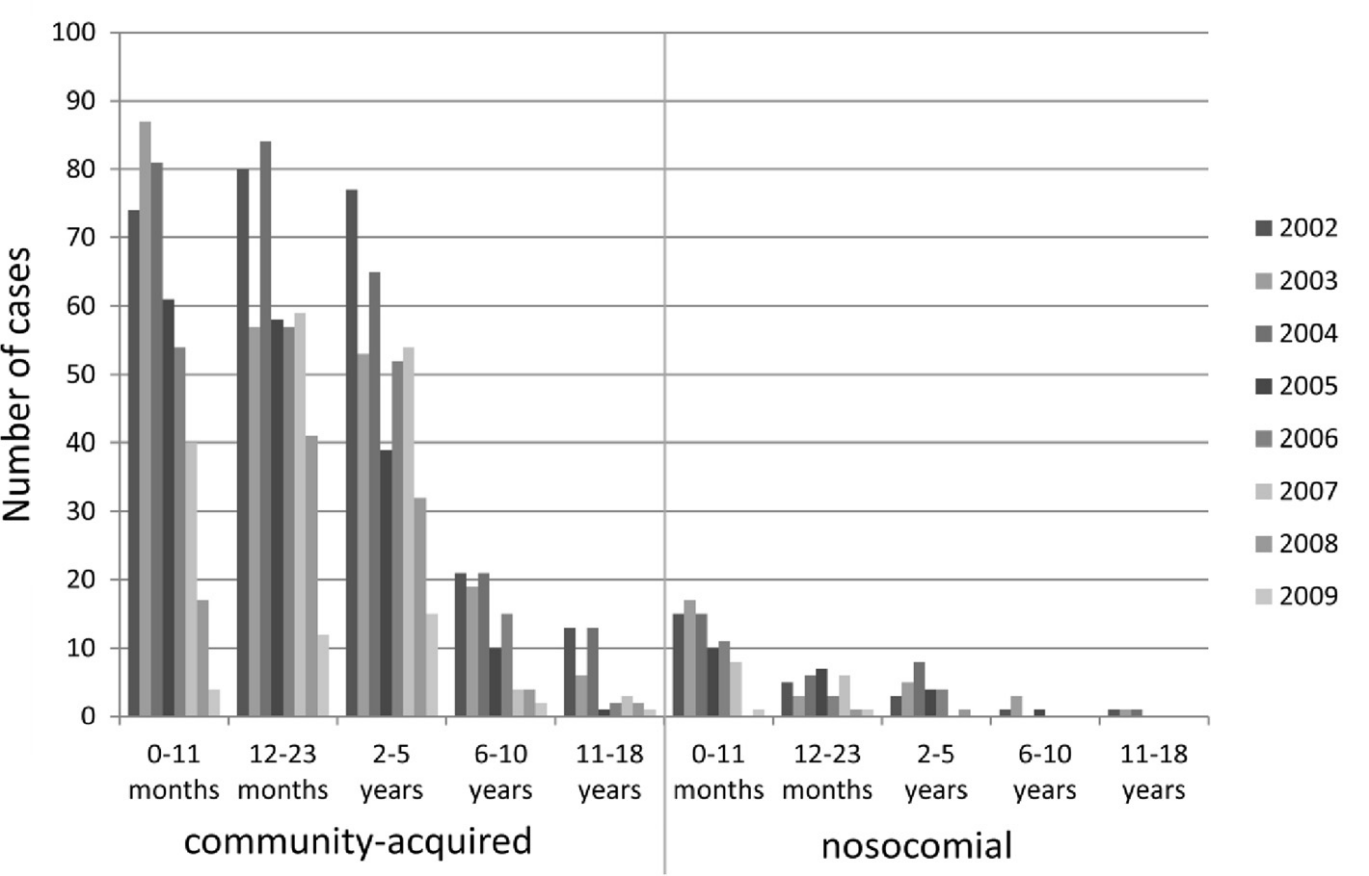
Numbers of community-acquired and nosocomial RV-GE cases.

**Figure 3:**
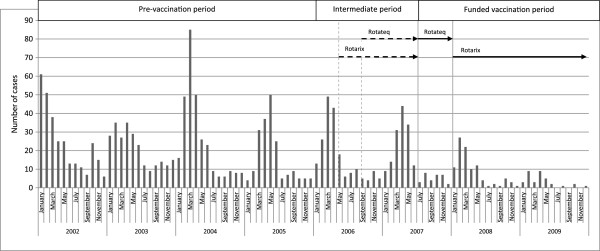
**Seasonal distribution and monthly numbers of hospitalized RV-GE cases.** In the study, the pre-vaccination period lasts from January 2002 to December 2005, the recommended and early funded vaccination period (intermediate period) from January 2006 to December 2007 and the funded vaccination period from January 2008 to December 2009. Rotarix was launched May 2006 and Rotateq was launched September 2006 (dotted lines and arrows). Rotateq was subsidized by the universal mass vaccination (UVM) program July to December 2007, Rotarix was subsidized by the UVM January 2008 to December 2009 (continuous lines and arrows) in Austria.

In the funded vaccination period, significantly fewer cases were nosocomial compared to the pre-vaccination period (p < 0.001) (Table 3). The absolute number of mean nosocomial cases per year decreased most strongly in the age group 0–11 months (Table 3). The proportional reduction was most pronounced in the age groups 0–11 months, 6–10 years and 11–18 years (Table 3, Figure 2). There was no change in seasonal peaks of RV-GE between the pre-vaccination and the other periods (Figure 3).

**Table 3:** Reduction of community-acquired and nosocomial RV-GE in age groups

**Age**	**Pre-vaccination period (2002–2005)**				**Intermediate period (2006–2007)**				**Funded vaccination period (2008–2009)**				**Reduction**^**a**^	
	**Total numbers (% of total numbers)**		**Numbers per year (mean; 95% CI)**		**Total numbers (% of total numbers)**		**% of pre-vaccination**		**Total numbers (% of total numbers)**		**Numbers per year (mean; 95% CI)**		**Decreased by (%)**	
	**CA**	**NO**	**CA**	**NO**	**CA**	**NO**	**CA**	**NO**	**CA**	**NO**	**CA**	**NO**	**CA**	**NO**
**0–11 mos**	303 (32.9)	57 (53.8)	75.8 (67.7–83.8)	14.3 (12.2–16.3)	94 (27.9)	19 (59.4)	47.0 (37.3–56.7)	9.5 (7.4–11.6)	21 (16.3)	1(25.0)	10.5 (1.5–19.5)	0.5 (-0.2–1.2)	86.1	96.5
**12–23 mos**	279 (30.4)	21 (19.8)	69.8 (57.8–81.8)	5.3 (4.3–6.3)	113 (33.5)	9 (28.1)	58.0 (56.6–59.4)	4.5 (2.4–6.6)	53 (41.1)	2 (50.0)	26.5 (6.4–46.6)	1.0 (0–1)	62.0	81.1
**2–5 yrs**	234 (25.4)	20 (18.9)	58.5 (46.3–70.8)	5.0 (3.8–6.2)	106 (31.5)	4 (12.5)	53.0 (51.6–54.4)	2.0 (-0.8–4.8)	46 (35.7)	1 (25.0)	23.5 (11.7–35.3)	0.5 (-0.2–1.0)	59.8	90.0
**6–10 yrs**	71 (7.7)	5 (4.7)	17.8 (13.9–21.6)	1.3 (0.6–2.0)	19 (5.6)	0	9.5 (1.9–17.1)	0	6 (4.6)	0	3.0 (1.6–4.4)	0	83.1	100
**11–18 yrs**	33 (3.6)	3 (2.8)	8.3 (3.6–12.9)	0.8 (0.5–1.1)	5 (1.5)	0	2.5 (1.8–3.2)	0	3 (2.3)	0	1.5 (0.8–2.2)	0	81.9	100
**All**	920 (100)	106 (100)	229.5 (196.7–262.3)	26.5 (24.1–29.0)	337 (100)	32 (100)	170.0 (160.7–178.6)	16.0 (13.2–18.8)	129 (100)	4 (100)	64.5 (20.8–108.2)	2.0 (0–2.0)	71.9	92.5

Abbreviations: *mos* months, *yrs* years, *CA* community-acquired, *NO* nosocomial, *CI* confidence interval.

^a^Reduction is calculated between mean numbers per year of the pre-vaccination period and the funded vaccination period (decrease in percentage).

In 2003 to 2005, 3 patients died because of nosocomial RV-GE due to deterioration of their underlying diseases and co-morbidity (tetralogy of Fallot; transplantation of the small intestine and secondary BSI with multi-organ failure; Rett-syndrome). No patient died because of RV-GE in the other periods.

Searching for co-infections, in the pre-vaccination period, at least one additional pathogen was detected in the stool samples of 41 (4.0%) out of 1026 patients with confirmed RV-GE (mean 10.3 cases per year; 95% CI 8.4–12.1) and in the stool samples of 13 (9.7%) out of 134 patients (mean 6.5 cases per year; 95% CI 3.7–9.3) in the vaccination period (p < 0.01). Overall, the most frequent co-infecting pathogens were adenovirus (36 cases), *Salmonella ssp*. (26 cases), norovirus (18 cases) and *Campylobacter* (12 cases).

Secondary BSI after RV-GE occurred in 20 cases (pre-vaccination period: 14 cases, 1.4% out of 1026 patients; intermediate period: 3 cases, 0.8% out of 372 patients; funded vaccination period: 3 cases, 2.2% out of 134 patients) (Table 1), with 14 cases (70%) found in patients with nosocomial RV-GE. All patients were younger than 6 years with a mean C-reactive protein at the occurrence of BSI specific symptoms of 5.3 mg/dl. The most frequently detected pathogens were *Staphylococcus aureus* (60.0%), followed by *Enterobacteriaceae*. RV viremia was reported in one case.

### Hospitalization rates, duration of hospital stay and cost calculations

Hospitalization rates were reduced from an annual mean of 55.3/10,000 person-years in the pre-vaccination period and 11.6/10,000 person-years in the intermediate period to 4.3/10,000 person-years in the funded vaccination period (reduction by 92.2%).

The mean duration of hospital stay for community-acquired RV-GE cases was shorter in the funded vaccination period compared to the pre-vaccination period (p < 0.01), which was most pronounced in the 6–10 years old children (p < 0.01) (Table 4). The mean duration of hospital stay for nosocomial RV-GE cases was 8.0 days (95% CI: 7.7–8.4) in the pre-vaccination period, 9.8 days (95% CI: 6.9–12.7) in the intermediate period and 13.3 days (95% CI: 5.8–20.7) in the funded vaccination period.

**Table 4:** Duration and total costs for all community-acquired RV-GE induced hospital stays in the study hospital

**Age**	**Pre-vaccination period (2002–2005)**		**Funded vaccination period (2008–2009)**		**Reduction**	
	**Duration of hospital stay (days) per year (mean; 95% CI)**	**Estimated mean of total costs per year (for patients/accompanying persons)**	**Duration of hospital stay (days) per year (mean; 95% CI)**	**Estimated mean total costs per year (patient/accompanying person)**	**Duration of hospital stay decreased by (%)**	**Costs per year decreased by (%)**
**0–11 mos**	3.4 (3.1–3.7)	224,216 €/ 7,865 €	3.0 (2.3–3.6)	29,295 €/ 1,087 €	11.8	86.9/86.2
**12–23 mos**	4.0 (3.6–4.3)	242,904 €/ 8,521 €	3.5 (3.4–3.5)	86,256 €/ 3,201 €	12.5	64.5/62.4
**2–5 yrs**	3.7 (3.6–3.9)	188,312 €/ 6,608 €	3.5 (3.1–3.8)	76,493 €/ 2,838 €	5.4	59.4/57.1
**6–10 yrs**	4.1 (3.7–4.4)	63,493 €/0	2.5 (1.8–3.2)	6,975 €/0	39.0	-
**11–18 yrs**	3.9 (2.8–4.9)	28,162 €/0	3.8 (2.7–4.8)	5,301 €/0	2.6	-
**All**	3.8 (3.5–4.1)	747,087 €/22,994 €	3.3 (2.7–3.8)	204,320 €/ 7,126 €	13.2	72.7/69.0

Calculation:

Pre-vaccination group: Estimated mean of total costs per year = annual mean number of patients with community-acquired RV-GE in the respective age group x mean duration of hospital stay x mean cost per patient/accompanying person.

Post-vaccination group: mean of total costs per year = annual mean number of patients with community-acquired RV-GE in the respective age group x mean duration of hospital stay x mean cost per patient/accompanying person.

In the funded vaccination period, total direct costs were reduced by 72.7% compared to the pre-vaccination period (p < 0.01) (Table 4). The reduction of direct costs for patients and accompanying persons was most pronounced in the age group 0–5 months.

## Discussion

The present study clearly demonstrates that UMV not only led to reduction of RV-GE-associated hospitalizations by 73.9%, but also to a pronounced reduction of nosocomial RV infections by 92.5%. The possible indirect effect of UMV on nosocomial RV infections may be an important aspect considering severe disease courses in hospitalized patients with co-morbidities. This was corroborated by the fact that mortality in our pre-vaccination cohort was attributed to deterioration of the underlying disease by nosocomial RV infection. A reduction of nosocomial RV infection was also seen in the US shortly after introduction of RV vaccination [32].

Also, in our study, secondary BSI was, in the majority of cases, linked to nosocomial RV-GE. In infants, RV infection has been described as a cause of pneumatosis intestinalis, hemorrhagic gastroenteritis, necrotizing enterocolitis and secondary BSI with mainly pathogens belonging to the intestinal microflora and the members of the *Enterobacteriaceae* family [24-29,33,34]. In our cohort, many of the 20 patients with secondary BSI were preterm infants or patients with compromised immune system and showed BSI due to *Staphylococcus aureus*. These findings allow us to hypothesize that not only intestinal mucosa dysfunction due to RV-GE promotes transition of intestinal bacteria, but that also a fatal combination of severe underlying diseases with dehydration and malnutrition in succession of RV-GE could have made patients more prone for secondary BSI caused also by non-intestinal bacteria. Although both RV vaccines cover the most important serotypes even in Austria [11], so far we are not able to know whether a shift to other RV serotypes will take place in the future causing inefficiency of the vaccine serotypes [35,36] and whether other gastroenteritis pathogens will take over.

The reduction of community-acquired hospitalized RV-GE cases found in our study confirms the data of recently published studies [5,11,12] which showed a significant reduction of RV cases reported by sentinel hospitals in the vaccination period in all age groups. In our study, the age group 0–11 months had the highest benefit of vaccination, highlighting the importance of starting the vaccination as early as possible [12]. A clear reduction in the older individuals indicates the presence of herd protection in the population by reduction of RV transmission [12,37,38]. However, also transmission of the attenuated RV types from vaccinated children to unvaccinated individuals may induce some immunity against RV in the unvaccinated older population [39-41].

In the US, a 50% decrease in RV-positive laboratory tests has been found after recommendation of RV vaccination for routine use in 2006, showing a delay in seasonal onset of the RV season 2007–2008 by 2–4 months [2]. These data are in contrast to our data and previous data from Austria [11] which could not detect a shift of RV-associated hospitalizations to later months. However, the findings of the US study are limited by missing data from the end of the RV season 2007–2008 and by the fact that RV was tested based on the discretion of the physicians and local policies.

In our cohort, we could demonstrate a reduction of mean annual real costs by 72.7% between the pre-vaccination and the funded vaccination period, although it was not possible to include indirect costs, such as work loss by parents staying with the hospitalized child and supervision of siblings staying at their own. Considering the direct and indirect costs of hospital admissions due to RV-GE, UMV programs have been shown to be cost-effective [42] and would lead to a reduction of costs for RV-GE-related hospitalizations/emergency visits by 83% and for medical consultations by 75% [43]. In our study, in the funded vaccination period, hospital stays were about half a day shorter than in the pre-vaccination period which may also be accounted for by milder disease courses [44].

Limitations of our study exist in the fact that only hospitalized cases were counted and that local health-seeking behaviors of the population and hospital-specific guidelines for admission may influence the hospitalization rates [3]. We here reported the results of a single, large, tertiary care center and might have consistently missed mild cases of RV-GE which were treated at home. One disadvantage of a single-center experience also exists for differences in incidence oscillations by seasonal forcing and demographic forcing that may cause different patterns of hospitalizations in different places. Certainly not all RV cases would have been detected and might have been influenced by different detection systems and coding practices. The detection system was changed in 2005 to a more sensitive method but with similar specificity. Additionally, the nature of the disease itself has to be taken into account, as wild-type RV infection does not prevent re-infection, or does not consequently enhance protection against recurrent infection [45-47]. However, the relative risk of re-infection is lower after one RV infection (0.62), two infections (0.4) or three subsequent infections (0.34) [47]. Subsequent infections are often subclinical or mild [47] and will not consequently lead to hospitalization of the children. Milder and asymptomatic cases will be less infectious, both as a result of reduced viral shedding rate and a shorter duration of infection. Thus, vaccination will cause fewer infections by making vaccinated individuals less able to become re-infected, if vaccination elicits the same immunological response as natural infections, and less able to transmit RV to others as predicted by transmission dynamic models [48]. In addition, also the numbers of older children may be underestimated, as most of them will suffer from mild infection without need for hospitalization [11]. Hospitalized re-infections were not assessed in our study, as double-counters were excluded to avoid biasing our results. Interestingly, in our study, 557 patients were double-counters, however, most of them were repeated detections of RV antigen in the stool samples during the same hospital stay or in immunocompromised patients showing a slow clearance from intestinal RV replication [41]. Unfortunately, due to the retrospective character of our study, the vaccination status was not sufficiently documented in the investigated patients, thus, despite being certainly of interest, break-through RV infections could not be assessed. Natural epidemiological oscillations are other factors which might have influenced our results. However, epidemiological studies since 1997 based on a sentinel system showed that fluctuations of RV-GE numbers were always less than the decrease observed since introduction of UMV [5]. An unrecognized change in the prevention of pathogen transmission was excluded in our study, as similar hygienic rules and introduction of nurses, parents and visiting persons were applied during the whole study period, interventions which have been shown to be crucial in reduction of transmission [20].

## Conclusions

In conclusion, UMV against RV may have contributed to the significant decrease of hospitalizations of RV-GE since 2008 [11,12] and, most important, to a reduction of nosocomial RV infections and RV-associated morbidity due to secondary BSI and reduced direct hospitalization costs. The reduction in nosocomial cases is an important aspect considering severe disease courses in hospitalized patients with co-morbidities and death due to nosocomial RV-GE.

## Competing interests

The authors declare that they have no competing interests.

## Authors’ contributions

MZ performed the data analysis, participated in writing of the manuscript and designed the figures and tables. SK performed the data acquisition and analysis. DO performed the laboratory tests, the data acquisition and participated in interpretation of the data. RW and AS participated in interpretation of the data and revised the manuscript critically for important intellectual content. PHE performed the laboratory tests and participated in interpretation of the data. MP designed and coordinated the study, performed the data analysis and wrote the manuscript. All authors gave final approval of the manuscript.

## Pre-publication history

The pre-publication history for this paper can be accessed here:

http://www.biomedcentral.com/1471-2334/13/112/prepub
